# *Burkholderia pseudomallei* in Colombia: Laboratory Approaches to Enhance Diagnostic Accuracy

**DOI:** 10.1155/ijm/7148191

**Published:** 2025-10-05

**Authors:** Soraya Morales-Lopez, Yeneiris Villero Wolf, Deyner Lechuga, Luis Caicedo, Yiceth Acosta Triana, Liliana Gomez, Luis Rodrigo Ramirez, Leandro Narvaez, Heidy Pinzon, Kelin Esquea, Rafael Barros, Pedro Martinez Ramos, Adriana Marin, Claudia Marcela Parra

**Affiliations:** ^1^Department of Microbiology, Nancy Florez Garcia Laboratories, Valledupar, Cesar, Colombia; ^2^Department of Microbiology, CINBIOS group, Universidad Popular del Cesar, Valledupar, Cesar, Colombia; ^3^Department of Bacteriology, CIENCIAUDES Group, Universidad de Santander, Valledupar, Cesar, Colombia; ^4^Department of Microbiology, Organización Humana Integral-OHI, Valledupar, Cesar, Colombia; ^5^Department of Microbiology, Luz Angely Nacimiento Laboratory, Valledupar, Cesar, Colombia; ^6^Department of Microbiology, Laboratorios Asociados, Valledupar, Cesar, Colombia; ^7^Department of Microbiology, Alta Complejidad del Caribe Clinic, Valledupar, Cesar, Colombia; ^8^Department of Microbiology, Laura Daniela Clinic, Valledupar, Cesar, Colombia; ^9^Department of Microbiology, Clínica del Cesar, Valledupar, Cesar, Colombia; ^10^Department of Microbiology, Microvidas Laboratories, Córdoba, Colombia; ^11^Department of Microbiology, Organización Clínica General del Norte, Barranquilla, Colombia; ^12^Department of Microbiology and Parasitology, Universidad Complutense de Madrid, Madrid, Spain

**Keywords:** Ashdown's agar, *Burkholderia cepacia*, *Burkholderia pseudomallei*, CHROMID Colistin, MALDI-TOF, melioidosis, Vitek 2 Compact

## Abstract

**Background:**

Melioidosis is a challenging disease to diagnose, and diagnostic complications can delay treatment, adversely impacting patient outcomes.

**Methods:**

Over a period of 1 year, 68 isolates, initially identified as *Burkholderia* spp. or oxidase-positive nonfermenting Gram-negative bacilli (excluding *Pseudomonas aeruginosa*), were collected from laboratories in three Colombian cities. Four commercial identification systems were employed. After recovery on blood agar, all strains were cultured on Ashdown's and CHROMID Colistin R media. Comparative identification using automated systems was performed, and definitive identification was achieved through multiplex PCR.

**Result:**

PCR identified three *Burkholderia pseudomallei* isolates and 59 *Burkholderia cepacia* complex isolates. The Microscan and Vitek 2 Compact systems successfully identified the *B. pseudomallei* isolates, whereas the Phoenix and MALDI-TOF Bruker systems did not. Ashdown's CHROM colistin media supported the growth of *B. pseudomallei* and various other genera and species. Species-level misidentifications were frequent.

**Conclusion:**

Due to limitations of commercial identification systems and the morphological similarities between species, the use of molecular tools or a combination of confirmatory tests is crucial for accurately diagnosing *B. pseudomallei* in Colombia.

## 1. Introduction

Melioidosis, a potentially fatal neglected tropical disease, is acquired by percutaneous inoculation, inhalation, or ingestion, and is commonly associated with individuals in close contact with water or soil. If left untreated, melioidosis carries a mortality rate of up to 95% [1]. The sole causative agent of melioidosis is now known to be *Burkholderia pseudomallei*, and its isolation from any clinical sample is diagnostic of infection [1–3].

The true incidence of melioidosis remains obscured in many tropical and developing countries due to a lack of diagnostic expertise and inadequate healthcare [4, 5]. In Colombia, the disease is endemic but underreported [4], although several cases have been documented in recent years [6, 7].

While *B. pseudomallei* grows on routine media, selective agar such as Ashdown's agar is essential to prevent bacterial overgrowth [3, 8]. Additionally, *B. pseudomallei* is often misidentified as *Burkholderia cepacia*, *Pseudomonas fluorescens*, *Pseudomonas aeruginosa*, or *Chromobacterium violaceum*, and in recent reports, *Acinetobacter*, even when using commercial detection systems. Such misidentifications may result in detrimental outcomes [1, 9–12].

Given the critical need for accurate diagnosis and reliable identification of *B. pseudomallei* in clinical settings, this study is aimed at assessing the accuracy of various phenotypic methods used in Colombia, along with selective agars, to offer recommendations for improving diagnostic accuracy.

## 2. Materials and Methods

Over a period of 1 year, isolates identified as belonging to the genus *Burkholderia* and any oxidase-positive Gram-negative bacillus, excluding *Pseudomonas aeruginosa*, were prospectively collected from 10 laboratories (same number of clinics) across three cities in the northern Colombian Caribbean region.

Each laboratory employed at least one of the following commercially available microbial identification systems: Vitek 2 Compact (bioMérieux France), MicroScan (Beckman Coulter, United States), Phoenix (Becton Dickinson, United States), or matrix-assisted laser desorption/ionization time of flight mass spectrometry, MALDI TOF Biotyper v3.1 (Bruker Daltonik GmbH, Bremen, Germany), and Vitek MS IVD (biomerieux, Marcy l'Etoile, France). The microbial identification systems used in this study were not selected by the research team but rather reflected the routine diagnostic platforms already in place at each participating clinical laboratory. Therefore, each site employed one or more of the available commercial systems (Vitek 2, MicroScan, Phoenix, MALDI-TOF Biotyper v3.1, or VITEK MS IVD), depending on local infrastructure and diagnostic capacity.

The isolates were handled in a biological safety cabinet with appropriate protection elements. After recovery on blood agar and Gram stain, they were streaked on Ashdown's agar (trypticase soy agar, 10 g/L; agar-agar, 15 g/L; crystal violet, 5 mg/L; neutral red, 50 mg/L; 4% glycerol stock solution, 100 mL/L; gentamicin, 4 mg/L; and double distilled water) [13]. The plates were incubated in ambient air at 37°C and monitored for up to 1 week. Positive growth was defined as the organism's ability to grow within inoculum or away from the initial streak [14]. *Enterococcus faecalis* ATCC 29212, *Escherichia coli* ATCC 21922, *Staphylococcus aureus* ATCC 25923, *Pseudomonas aeruginosa* ATCC 27853, and *Candida albicans* ATCC 10231 were used to evaluate the medium's specificity.

Next, direct streaking on CHROMID Colistin R agar plates (bioMérieux SA, Marcy l'Etoile, France) was performed to detect Colistin resistance using a primary inoculation on brain heart infusion broth and incubation at 35°C ± 2°C, 4–5 h with a disc of 10 *μ*L colistin. Results were interpreted after 18–24 h of aerobic incubation at 35°C–37°C [15, 16]. *E. faecalis* ATCC 29212 and *C. albicans* ATCC 10231 were used as negative quality control. These strains were first inoculated into BHI broth and incubated overnight at 37°C, and then, a loopful was streaked onto CHROMID agar plates.

For molecular identification, DNA was extracted using the Gene Jets Genomic DNA purification kit (Thermo Scientific, cat K0722). A multiplex PCR assay, using four primer sets (32F and 32R; 51F and 51R; 71F and 71R; and 16F and 16R), was conducted for the rapid identification and differentiation of *B. pseudomallei, Burkholderia thailandensis, Burkholderia mallei,* and *B. cepacia* complex was used [17]. The PCR was performed in a 30-*μ*L reaction volume containing 6 *μ*L of genomic DNA; 0.2 mM of each dNTP; 1X Pol Buffer C; 1.5 mM MgCl_2_; 0.75 unit MyTaq DNA Polymerase (Promega, cat M3001, United States); 0.2 *μ*M primers 16F, 16R, 32F, 32R, 71F, and 71R; and 0.3 *μ*M primers 51F and 51R. The amplification conditions were initial denaturation at 95°C for 5 min, followed by 35 cycles at 95°C for 30 s, 59°C for 45 s, and 72°C for 45 s, with a final extension at 72°C for 5 min. The reaction was carried out using a 96-well Thermal Cycler (Veriti 96, Applied Biosystems by Thermo Fisher Scientific, United States), and the products were analyzed by electrophoresis on a 1.5% agarose gel (wt/vol) at 100 V for 60 min, stained with ethidium bromide. The presence of specific fragments was used to interpret the multiplex PCR assay: three fragments for *B. pseudomallei* (321 bp, 516 bp, and 709 bp), two for *B. mallei* (516 bp and 709 bp), and one each for *B. thailandensis* (709 bp) and *B. cepacia* complex (560 bp). PCR assays targeting species-specific genes were performed as detailed in Table 1. Control DNAs from *B. pseudomallei* and *B. cepacia* were provided by the Instituto Nacional de Salud of Colombia.

### 2.1. Ethical Consideration

This study was approved by the Research Ethics Committee of Universidad Popular del Cesar (UPC), in accordance with the ethical guidelines established by Resolution 8430 of 1993 from the Colombian Ministry of Health. The study did not involve human subjects, patient data, or clinical records.

## 3. Results

A total of 68 bacterial isolates were collected and identified as *Burkholderia cenocepacia* (2), *Burkholderia lata* (1), *Burkholderia contaminans* (2), *B. thailandensis* (2), *B. pseudomallei* (1), *B. cepacia* (59), *and Chryseobacterium indologenes* (1) from various clinical samples, including blood, urine, ulcer, marrow, tissue, eschar, and secretion (Table 2). Standard laboratory procedures served as the basis for the reported laboratory identification.

All isolates produced white to cream-colored colonies on blood agar, except for the *C. indologenes* isolate, which produced yellow colonies. Gram staining revealed that all isolates appeared as short gram-negative bacilli without bipolar staining.

**Figure 1:**
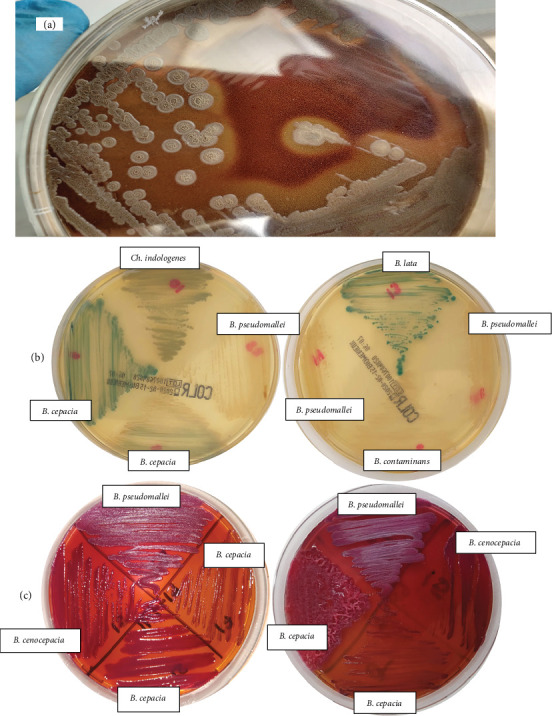
(a) *B. pseudomallei* on blood agar, showing dry and wrinkled morphology after 3 days of incubation. (b) Growth of colistin-resistant isolates on CHROMagar R COL (some isolates exhibit green colonies). (c) Colony morphologies of various *Burkholderia* strains on Ashdown's agar. *B. pseudomallei* isolates (top) display a distinct metallic sheen.

Then, 11 isolates—*B. cepacia* (6), *B. thailandensis* (1), *C. indologenes* (1), *B. cenocepacia* (1), *B. contaminans* (1), and *B. pseudomallei* (1)—successfully grew on Ashdown´s agar, with four of them (*B. cepacia, B. thailandensis* (2), *and B. pseudomallei*) producing a characteristic metallic sheen. On CHROMID Colistin agar, 24 isolates demonstrated growth, including *C. indologenes* (1) and 15 isolates for the *B. cepacia* complex, which unexpectedly formed with green colonies (Figure 1 and Table 2).

**Figure 2:**
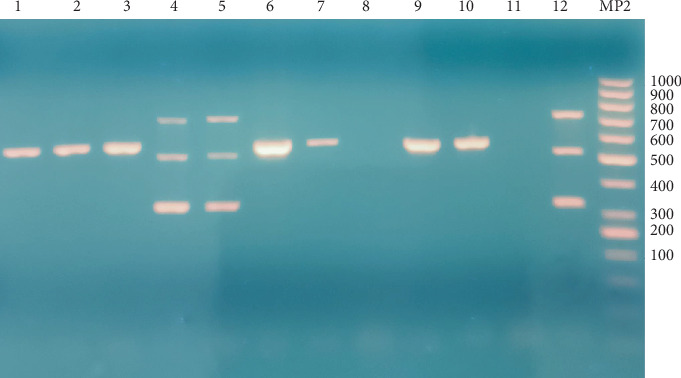
Multiplex PCR detection of *B. mallei*, *B. pseudomallei*, *B. thailandensis*, and *B. cepacia*. Lanes: MP2 DNA ladder (100 bp); Lanes 1, 2, 3, 6, 7, 9, and 10: *B. cepacia* complex isolates; Lanes 4, 5, and 12: *B. pseudomallei* isolates; Lane 8: *Chryseobacterium indologenes* isolate; Lane 11: *Stenotrophomonas maltophilia* isolate.

PCR analysis identified 59 isolates with band patterns consistent with *B. cepacia* complex, while three isolates matched *B. pseudomallei* (Figure 2). No *B. mallei* or *B. thailandensis* isolates were detected. In six cases, no recognizable banding patterns were found, leading to identification through MALDI-TOF instruments (Bruker Daltonik GmbH, Bremen, Germany and Vitek MS (biomerieux, Marcy l'Etoile, France). These isolates were subsequently identified as *Serratia marcescens* (4), *C. indologenes* (1), and *Stenotrophomonas maltophilia* (1).

Based on these new findings, identification using an alternative commercial system to the one initially employed was also performed, and the results are summarized in Table 2.

## 4. Discussion

Identifying *B. pseudomallei* in clinical microbiology laboratories is challenging, especially in areas where clinical suspicion is low. Therefore, in endemic regions, any oxidase-positive Gram-negative bacillus that is not *Pseudomonas aeruginosa* should be considered as a potential *B. pseudomallei* [18].

In this study, none of the isolates exhibited bipolar staining, which is typically due to the central accumulation of polyhydroxybutyrate granules. However, Gram stain appearance alone is insufficient for a presumptive diagnosis [19].

Since culture remains the diagnostic gold standard, the isolation of *B*. *pseudomallei* has been enhanced by the use of selective media such as Ashdown's agar [20]. Ashdown's agar [13] is the most widely used selective medium in endemic countries [3]. Other selective media, such as *B. pseudomallei* selective agar, Francis medium agar, and *B. cepacia* selective agar, have been developed to support the selective growth of *Burkholderia* spp. [1, 21, 22]. However, none of these selective media are commercially available in Colombia. Additionally, the latex agglutination test, another useful screening tool for the identification of *B. pseudomallei*, is also unavailable in the country [20].

In this study, Ashdown's medium not only supported the growth of three *B. pseudomallei* isolates but also allowed the growth of organisms outside the genus, such as *C. indologenes*, and several *B. cepacia* complex isolates. Similar observations were reported by Edler et al. [8], who found that non-target organisms, including other non-fermentative rod-shaped bacteria and yeasts, could grow on Ashdown's agar. Conversely, there are reports of *B. pseudomallei* being isolated without growth on Ashdown's agar [20].

In our study, the presence of a metallic sheen characteristic on Ashdown's medium was limited to three *B. pseudomallei* isolates and one isolate identified as *B. cepacia* using Phoenix, which was later reidentified as *B. metallica* using Bruker's MALDI-TOF. Additionally, CHROMID Colistin R agar, which is available in Colombia, was evaluated for its ability to screen colistin-resistant organisms. This agar is primarily used for surveillance and recovery of colistin-resistant bacteria and has demonstrated 100% category agreement with the broth microdilution reference method in *Enterobacteriaceae* [23]. Similar to Ashdown's agar, ChromID Colistin R agar allowed the growth of all *B. pseudomallei* isolates, as well as other species within and outside the *Burkholderia* genus.

To our knowledge, this is the first study in Colombia that utilized Ashdown's and the first to employ CHROMID Colistin R agar to assess colistin resistance in *Burkholderia* spp. These findings contribute to establishing a presumptive workflow for the rapid identification of *B. pseudomallei* in clinical laboratories for the diagnosis of melioidosis.

Commercial identification systems often show variability in their performance and may fail to accurately differentiate between *B. pseudomallei* and closely related species [18, 20, 22, 24, 25]. In this study, the Microscan and Vitek 2 systems successfully identified the *B. pseudomallei* isolates, while the Phoenix and MALDI-TOF systems misidentified the isolates as *B. cepacia* and *B. thailandensis*, respectively.

Previous studies have reported the limitations of the Vitek 2 in correctly identifying *B. pseudomallei* [20]. Some authors have also noted instances where Vitek 2 failed to identify *B. pseudomallei*, with subsequent MALDI-TOF MS misidentifying the organism as *B. cepacia* or *Ochrobactrum anthropi.* In such cases, molecular testing revealed that the isolates belonged to the *B. cenocepacia* or *B. cepacia* complexes. This misidentification of the *B. cepacia* complex as the *B. pseudomallei* complex poses significant public health risks [10].

In this investigation, one strain was identified as *S. maltophilia* by both MALDI-TOF systems (Bruker and Vitek MS) but showed no banding pattern via PCR. This strain had previously been identified within the *B. cepacia* complex. These results align with findings reported by other authors [10].

Currently, the BD Phoenix system (Becton Dickinson, Sparks, MD), which is widely used in Colombia, lacks *B. pseudomallei* in its database, leading to frequent misidentifications of this organism as *B. cepacia*. [26, 27]. Although MALDI-TOF is a revolutionary and rapid tool for pathogen identification, accurate identification of *B. pseudomallei* requires optimized in-house databases with added reference spectra [18, 26, 28–30].

In this study, MALDI-TOF MS biotyper v3.1 (Bruker Daltonic) identified *B. thailandensis* with a score range between 1890 and 1899, which is a valid identification at the genus level. The accompanying report highlighted the close relationship between *B. pseudomallei* and *B. thailandensis*, suggesting further testing and a clinical, epidemiological, and geographical assessment before ruling out *B. pseudomallei* as the causative agent. One of the three described *B. pseudomallei* isolates was later confirmed by whole-genome sequencing, as reported by other authors [31].

*B. pseudomallei* is currently not included in the in vitro diagnostic (IVD) database of the MALDI Biotyper v3.1 (Bruker Daltonik GmbH) and the Vitek MS (bioMérieux) v 3.2. However, *B. pseudomallei* is present in the research-use-only (RUO) databases of both systems [32].

The RUO, super spectra (SS), or security-relevant (SR) libraries were not employed in this study. The RUO database is intended for investigational purposes and is not approved for routine clinical diagnostics. The SS or SR library, which includes highly pathogenic organisms such as *B. pseudomallei*, is not part of the standard MALDI-TOF identification workflow and requires special authorization due to biosecurity considerations. Therefore, the identification of *B. pseudomallei* by MALDI-TOF was limited by its absence in the Biotyper v3.1 (Bruker) or IVD (bioMérieux) libraries and would only have been possible where RUO or SR spectra were available.

While molecular methods for species identification have been described, their utility has been limited to research and reference laboratories due to genetic variability, recombination, and lack of validation in large data sets [2, 33, 34]. In this study, the identifications obtained by PCR were consistent with those achieved through the Vitek 2 and Microscan commercial methods. Cases where PCR amplification failed corresponded to isolates that fell outside the *Burkholderia* genus, such as *Serratia* or *Stenotrophomonas*, as confirmed by mass spectrometry–based commercial methods.

Misidentification of *B. pseudomallei* using routine clinical laboratory methods can lead to incorrect treatment and unfavorable patient outcomes. Therefore, phenotypic methods—including the simple combination of oxidase positive, gentamicin resistant, amoxicillin–clavulanate susceptible, use of Ashdown's agar, Colistin R agar, and automated identification systems—are critical tools in diagnosing melioidosis and monitoring *B. pseudomallei* in Colombia. However, definitive identification should always be confirmed by PCR for accurate diagnosis and proper patient management.

## 5. Conclusions

This study explored different diagnostic methods for identifying *B. pseudomallei* using available equipment in Colombia. Although the number of isolates was limited, it provides valuable insights into the detection frequency of *B. pseudomallei* from clinical oxidase positivity nonfermenting Gram-negative bacilli other than *Pseudomonas aeruginosa* and *Burkholderia* species.

Ashdown's medium has proven to be a reliable selective medium and should be routinely used in clinical laboratories when melioidosis is suspected. However, since both Ashdown's and CHROMID Colistin R media also support the growth of various nontarget microorganisms, their use must be paired with confirmatory tests to ensure the accurate screening of melioidosis.

Given the limitations in the existing databases, laboratories must remain vigilant to the risk of *B. pseudomallei* misidentification by automated identification systems. Mass spectrometry may serve as a useful confirmatory tool, provided that validated reference spectra are available. Ensuring access to comprehensive and accurate MALDI-TOF libraries is essential for the reliable identification of *B. pseudomallei*.

Detailed methodologies and diagnostic algorithms aligned with this approach are outlined in the Melioidosis Reference Manual available at http://www.melioidosis.info [35]. When automated systems prove insufficient, molecular testing remains an important tool for correctly identifying *B. pseudomallei*, emphasizing the need for a comprehensive, multifaceted diagnostic approach to melioidosis.

## Figures and Tables

**Table 1:** Target genes used for PCR identification of *Burkholderia* species.

***Burkholderia* species**	**Target gene**	**Primer**	**Sequence 5**⁣′** to 3**⁣′	**Amplicon size (bp)**
*B. pseudomallei, B. mallei, B. thailandensis.*	Putative sugar binding protein	71F	AGCTCGCAGATGA ACTGGAT	709
		71R	GCTGATCTGTTT GCTGCTGTA	
*B. pseudomallei, B. mallei, B. cepacia complex.*	Hypothetical protein	51F	CCCAATCAGACC ACGATTATT	516
		51R	GTTCAACGCGC CTTTATTGT	
*B. pseudomallei.*	Putative outer membrane protein	32F	TCTGCTTCATGC TGTTTTCA	321
		32R	GGCGCTGATATA CAGTGTCTC	

**Table 2:** Results of identification of 68 isolates of *Burkholderia* spp. or oxidase-positive Gram-negative bacillus not *Pseudomonas aeruginosa* from clinical specimens.

**ID**	**Identification**	**Source**	**System ID**	**AS color**	**Ashdown growth**	**CHROMID growth**	**PCR band**	**Id PCR**	**Additional commercial system for identification**
BUR 1	*B. cepacia*	Blood	Microscan	White	Pos	+ (green)	560	BCC	*B. cepacia* (MALDITOF Bruker)
BUR 2	*B. cepacia*	Urine	Phoenix	Cream	−	−	560	BCC	*B. contaminans* (MALDITOF MS Vitek)/*B cepacia* (MALDITOF Bruker)
BUR 3	*B. cepacia*	Blood	Maldi Bruker	Cream	−	+	560	BCC	
BUR 4	*B. cepacia*	Blood	Maldi Bruker	Cream	−	+	560	BCC	
BUR 5	*B. cepacia*	Blood	Maldi Bruker	Cream	Pos	+ (green)	560	BCC	
BUR 6	*B. cepacia*	Wound	Microscan	White	−	+ (green)	560	BCC	*B. cepacia* (MALDITOF Bruker)
BUR 7	*B. cenocepacia*	Blood	Maldi Bruker	Cream	−	−	560	BCC	*B. contaminans (*MALDITOF MS Vitek)
BUR 8	*B. cepacia*	Wound	Phoenix	Cream	−	−	560	BCC	*B. cepacia* (MALDITOF Bruker)
BUR 9	*B. cepacia*	Orotracheal tube	Phoenix	Cream	Pos	+ (green)	560	BCC	*B. cepacia* (MALDITOF Bruker)
BUR 10	*C. indologenes*	Blood	Phoenix	Dark Yellow	Pos	+ (green)	No band	—	*C. Indologenes (*MALDITOF MS Vitek)/MALDITOF Bruker)
BUR 11	*B. cepacia*	Wound	Phoenix	Metalic sheen	−	−	560	BCC	*B. metallica (*MALDITOF Bruker)
BUR 12	*B. cepacia*	Orotracheal tube	Phoenix	Cream	Pos	+ (green)	560	BCC	*B. cepacia* (MALDITOF Bruker)
BUR 13	*B. lata*	Blood	Maldi Bruker	Cream	−	+ (green)	560	BCC	
BUR 14	*B. thailandensis*	Blood	Maldi Bruker	Metalic sheen	Pos	+	321, 516, 709	*B. pseudomallei*	*B. pseudomallei* (Vitek 2)
BUR 15	*B. cenocepacia*	Blood	Maldi Bruker	White	Pos	+ (green)	560	BCC	
BUR 16	*B. contaminans*	Ulcer	Vitek 2	Cream	—	+	560	BCC	*B. contaminans (*MALDITOF MS Vitek)/*B cepacia* (Phoenix)
BUR 17	*B. cepacia*	Orotracheal tube	Microscan	Cream	Pos	+ (green)	No band	—	*S. maltophilia (*MALDITOF MS Vitek/MALDITOF Bruker)
BUR 18	*B. contaminans*	Vagina	Microscan	Cream	Pos	+ (green)	560	BCC	*B cepacia* (Phoenix/MALDITOF Bruker)
BUR 19	*B. cepacia*	Wound	Maldi Bruker	Cream	−	−	560	BCC	
BUR 20	*B. cepacia*	Wound	Phoenix	Cream	−	−	560	BCC	*B. cepacia* (MALDITOF Bruker)
BUR 21	*B. cepacia*	Sore	Phoenix	White	−	−	560	BCC	*B. cepacia* (MALDITOF Bruker)
BUR 22	*B. pseudomallei*	Blood	Microscan	Metalic sheen	Pos	+	321, 516, 709	*B. pseudomallei*	*B. pseudomallei* (Vitek 2)/*B. thailandensis* (MALDITOF Bruker)
BUR 23	*B. thailandensis*	Blood	Maldi Bruker	Metalic sheen	Pos	+	321, 516, 709	*B. pseudomallei*	*B. pseudomallei* (Vitek 2)
BUR 24	*B. cepacia*	Urine	Phoenix	White	−	−	560	BCC	*B. cepacia* (MALDITOF Bruker)
BUR 25	*B. cepacia*	Urine	Phoenix	Cream	−	−	560	BCC	*B. cepacia* (MALDITOF Bruker)
BUR 26	*B. cepacia*	Blood	Phoenix	Cream	−	−	560	BCC	*B. cepacia* (MALDITOF Bruker)
BUR 27	*B. cepacia*	Penis discharge	Phoenix	Cream	−	−	560	BCC	*B. cepacia* (MALDITOF Bruker)
BUR 28	*B. cepacia*	Breast discharge	Phoenix	Cream	−	−	560	BCC	*B. contaminans* (MALDITOF MS Vitek)/*B. cepacia* (MALDITOF Bruker)
BUR 29	*B. cepacia*	Wound	Phoenix	Cream	−	−	560	BCC	*B. cepacia* (MALDITOF Bruker)
BUR 30	*B. cepacia*	Tissue	Phoenix	Cream	−	−	560	BCC	*B. cepacia* (MALDITOF Bruker)
BUR 31	*B. cepacia*	Orotracheal tube	Microscan	Cream	−	−	560	BCC	*B. cepacia* (MALDITOF Bruker)
BUR 32	*B. cepacia*	Wound	Phoenix	Cream	−	−	560	BCC	*B. lata* (MALDITOF Bruker)
BUR 33	*B. cepacia*	Urine	Phoenix	Cream	−	−	560	BCC	*B. cepacia* (MALDITOF Bruker)
BUR 34	*B. cepacia*	Wound	Microscan	Cream	−	−	560	BCC	*B. cepacia* (MALDITOF Bruker)
BUR 35	*B. cepacia*	Orotracheal tube	Phoenix	Cream	−	−	560	BCC	*B. cepacia* (MALDITOF Bruker)
BUR 36	*B. cepacia*	Wound	Phoenix	Cream	−	−	560	BCC	*B. cepacia* (MALDITOF Bruker)
BUR 37	*B. cepacia*	Blood	Phoenix	Cream	−	−	560	BCC	*B. cepacia* (MALDITOF Bruker)
BUR 38	*B. cepacia*	Urine	Microscan	Cream	−	+ (green)	560	BCC	*B. cepacia* (MALDITOF Bruker)
BUR 39	*B. cepacia*	Blood	Microscan	Cream	−	−	560	BCC	*B. lata* (MALDITOF Bruker)
BUR 40	*B. cepacia*	Wound	Phoenix	Cream	−	−	560	BCC	*B. cepacia* (MALDITOF Bruker)
BUR 41	*B. cepacia*	Wound	Phoenix	Cream	−	−	560	BCC	*B. cepacia* (MALDITOF Bruker)
BUR 42	*B. cepacia*	Blood	Phoenix	Cream	−	−	560	BCC	*B. cepacia* (MALDITOF Bruker)
BUR 43	*B. cepacia*	Orotracheal tube	Phoenix	Cream	−	−	560	BCC	*B. cepacia* (MALDITOF Bruker)
BUR 44	*B. cepacia*	Orotracheal tube	Phoenix	Cream	−	−	560	BCC	*B. cepacia* (MALDITOF Bruker)
BUR 45	*B. cepacia*	Orotracheal tube	Phoenix	Cream	−	+	No band	—	*S. marcescens* (Vitek 2/MALDITOF Bruker)
BUR 46	*B. cepacia*	Wound	Phoenix	Cream	−	−	No band	—	*S. marcescens* (Vitek 2/MALDITOF Bruker)
BUR 47	*B. cepacia*	Wound	Phoenix	Cream	−	+ (green)	No band	—	*S. marcescens* (Vitek 2/MALDITOF Bruker)
BUR 48	*B. cepacia*	Wound	Phoenix	Cream	−	+ (green)	No band	—	*S. marcescens* (Vitek 2/MALDITOF Bruker)
BUR 49	*B. cepacia*	Wound	Phoenix	Cream	−	+ (green)	560	BCC	*B. cepacia* (MALDITOF Bruker)
BUR 50	*B. cepacia*	Sore	Phoenix	Cream	−	+ (green)	560	BCC	*B. cepacia* (MALDITOF Bruker)
BUR 51	*B. cepacia*	Sore	Phoenix	Cream	−	−	560	BCC	*B. cepacia* (MALDITOF Bruker)
BUR 52	*B. cepacia*	Tissue	Phoenix	Cream	−	−	560	BCC	*B. cepacia* (MALDITOF Bruker)
BUR 53	*B. cepacia*	Blood	Phoenix	Cream	−	−	560	BCC	*B. cepacia* (MALDITOF Bruker)
BUR 54	*B. cepacia*	Blood	Phoenix	Cream	−	−	560	BCC	*B. cepacia* (MALDITOF Bruker)
BUR 55	*B. cepacia*	Blood	Phoenix	Cream	−	−	560	BCC	*B. cepacia* (MALDITOF Bruker)
BUR 56	*B. cepacia*	Blood	Phoenix	Cream	−	−	560	BCC	*B. cepacia* (MALDITOF Bruker)
BUR 57	*B. cepacia*	Tissue	Phoenix	Cream	−	−	560	BCC	*B. cepacia* (MALDITOF Bruker)
BUR 58	*B. cepacia*	Orotracheal tube	Phoenix	Cream	−	−	560	BCC	*B. cepacia* (MALDITOF Bruker)
BUR 59	*B. cepacia*	Orotracheal tube	Phoenix	Cream	−	−	560	BCC	*B. cepacia* (MALDITOF Bruker)
BUR 60	*B. cepacia*	Vagina	Phoenix	Cream	−	−	560	BCC	*B. cepacia* (MALDITOF Bruker)
BUR 61	*B. cepacia*	Vagina	Phoenix	Cream	−	−	560	BCC	*B. cepacia* (MALDITOF Bruker)
BUR 62	*B. cepacia*	Orotracheal tube	Phoenix	Cream	−	−	560	BCC	*B. cepacia* (MALDITOF Bruker)
BUR 63	*B. cepacia*	Orotracheal tube	Phoenix	Cream	−	−	560	BCC	*B. cepacia* (MALDITOF Bruker)
BUR 64	*B. cepacia*	Wound	Phoenix	Cream	−	+	560	BCC	*B. cepacia* (MALDITOF Bruker)
BUR 65	*B. cepacia*	Wound	Phoenix	Cream	−	−	560	BCC	*B. cepacia* (MALDITOF Bruker)
BUR 66	*B. cepacia*	Urine	Phoenix	Cream	−	−	560	BCC	*B. cepacia* (MALDITOF Bruker)
BUR 67	*B. cepacia*	Wound	Phoenix	Cream	−	+ (green)	560	BCC	*B. cepacia* (MALDITOF Bruker)
BUR 68	*B. cepacia*	Urine	Microscan	Cream	Pos	−	560	BCC	*B. cepacia* (MALDITOF Bruker)

Abbreviation: BCC, *Bulkholderia cepacia* complex.

## Data Availability

Data are available on request from the authors.
